# Identification of a Ferroptosis-Related Signature Associated with Prognosis and Immune Infiltration in Adrenocortical Carcinoma

**DOI:** 10.1155/2021/4654302

**Published:** 2021-07-20

**Authors:** Xi Chen, Lijun Yan, Feng Jiang, Yu Lu, Ni Zeng, Shufang Yang, Xianghua Ma

**Affiliations:** ^1^Department of Endocrinology, The First Affiliated Hospital of Nanjing Medical University, Nanjing 210029, China; ^2^Department of Endocrinology, Taizhou Clinical Medical School of Nanjing Medical University (Taizhou People's Hospital), Taizhou 225300, China; ^3^Department of Hepatology, Nantong Third People's Hospital Affiliated to Nantong University, Nantong 226000, China; ^4^Department of Neonatology, Obstetrics and Gynecology Hospital of Fudan University, Shanghai 200011, China; ^5^Department of Dermatology, Affiliated Hospital of Zunyi Medical University, Zunyi 563000, China; ^6^Department of Nutriology, The First Affiliated Hospital of Nanjing Medical University, Nanjing 210029, China

## Abstract

Adrenocortical carcinoma (ACC) is a rare malignant tumor with poor prognosis. Ferroptosis, a new form of cell death, differs from other forms of cell death and plays a vital role in tumor progress. Our study aimed to establish a ferroptosis-related signature with prognostic value in ACC. RNA-seq data and corresponding clinical characteristics for ACC were downloaded from TCGA and GEO databases. Genes included in ferroptosis risk signature were assessed by univariable and multivariable Cox regression analysis as well as lasso regression analysis. The prognostic value of the ferroptosis risk signature was assessed using K-M and ROC curves. Furthermore, we performed GSEA to discover the enriched gene sets in high-risk group. Additionally, TIMER website was applied to detect a possible connection between the signature and immune cells infiltration. ssGSEA was performed to evaluate scores of immune cells and immune-related pathways in two groups. A ferroptosis signature comprised of six genes (SLC7A11, TP53, HELLS, ACSL4, PCBP2, and HMGB1) was constructed to predict prognosis and reflect the immune infiltration in ACC. Patients in high-risk group were inclined to have worse prognosis. The ferroptosis model performed well in predicting prognosis and could be served as an independent indicator in ACC. GSEA revealed that gene sets correlated with biological processes including cell cycle, DNA replication, base excision repair, and P53 signaling pathway were highly enriched in high-risk group. In addition, we discovered that the expressional levels of hub genes were linked to six immune cells' infiltration in ACC tumor. ssGSEA revealed that contents of most immune cells significantly decreased in the high-risk group. In conclusion, the novel ferroptosis risk signature could be useful in predicting prognosis and reflecting immune infiltration in ACC. It also brings us new insights into the possible value of targeting ferroptosis during the therapy of ACC.

## 1. Introduction

Adrenocortical carcinoma (ACC) is a rare and aggressive malignant tumor derived from adrenal cortex with dismal prognosis [1]. Although it is advantageous for ACC patients to receive complete surgical resection or treatment with mitotane, the 5-year survival rate is less than 40% [2, 3]. Meanwhile, prognosis varies based on age, scope of surgery, mitotic intensity, and hormone secretion. The present tumor, lymph node, and metastasis (TNM) classification method is unreliable in predicting prognosis owing to the heterogeneous features of ACC patients. It is challenging to make accurate prediction for ACC patients because of the diverse pathogenic factors, high heterogeneity, and poor prognosis. Hence, it is imperative to identify more effective biomarkers for predicting prognosis of ACC patients.

Ferroptosis is a novel iron-dependent form of regulated cell death along with iron accumulation and lipid peroxidation [4]. In the aspect of morphology, biochemistry, and genetics, ferroptosis differs from other forms of cell death such as apoptosis, necroptosis, autophagy, and pyroptosis [5]. Emerging researches demonstrated that ferroptosis is implicated in neurous system disorders and plenty of cancers [6, 7]. Several research studies have recently mined the online databases for identifying prognostic signatures based on ferroptosis-related genes in diverse cancers. Luo et al. produced a new ferroptosis-related signature that may predict prognosis in uveal melanoma patients [8]. For predicting the prognosis of low-grade gliomas, Zheng et al. constructed a risk signature that included 12 ferroptosis-associated genes [9]. However, no research has yet determined whether ferroptosis-related genes are linked to ACC patients' prognosis.

Firstly, we downloaded the mRNA expression data and relevant clinical information of ACC patients from public datasets. Then, based on the ferroptosis-related genes associated with overall survival (OS) from The Cancer Genome Atlas (TCGA) cohort, we developed a prognostic risk signature and validated it in Gene Expression Omnibus (GEO). We further performed Gene Set Enrichment Analysis (GSEA) to explore the underlying mechanisms. Finally, we evaluated the potential associations between the prognostic genes and immune cells based on the Tumor Immune Estimation Resource (TIMER).

## 2. Methods

### 2.1. Data Collection

As a training set, TCGA database was applied to collect the mRNA expression and related clinicopathological data of 79 ACC patients. In addition, 21 ACC patients with survival information from GEO database (GSE19750) were retrieved as a validation set. Supplementary Material Table S1 listed the detailed clinical information. A list of 130 ferroptosis-associated genes was downloaded from GeneCards detailed in Table S2. No ethical approval was required because the data we utilized were obtained from public databases.

### 2.2. Establishment and Validation of a Prognostic Ferroptosis Risk Signature

To construct a ferroptosis risk signature in ACC patients, univariate Cox regression analysis was applied to assess the association between ferroptosis-associated genes and OS. PPI network diagram of the candidate prognostic ferroptosis-related genes was drawn using STRING online database to explore the relationships between these genes. Ferroptosis-related genes with a *p* value < 0.05 in univariate analysis were considered as candidate genes and recruited into lasso-penalized Cox regression analysis to narrow the gene extent with independent prognostic value. Further multivariate Cox regression analysis was then used to eliminate the possible interaction among the candidate genes and obtain the coefficients. The risk score value of each patient was calculated by the following formula:

(1) $$\begin{array}{c} \text{risk score}=\sum_{i=1}^{n}\left(\text{coef mRNAi}*\text{expression of mRNAi}\right). \end{array}$$

Coef was the coefficient calculated by multivariable Cox regression. Risk score was calculated for each individual, and total patients in the TCGA and GEO databases were allocated into high- and low-risk groups according to the median risk score value. PCA and t-SNE were adopted to explore whether the risk model had reliable clustering ability. Kaplan–Meier (K-M) curves were generated to compare the survival difference in two groups. Moreover, receiver operating characteristic (ROC) curve and area under the ROC curve (AUC) were applied to evaluate the prognostic value of risk signature for OS in ACC patients. Furthermore, we performed univariate and multivariate Cox analysis to determine whether the risk score could serve as an independent factor for OS. We further applied GEO data to verify the above results through the same methods.

### 2.3. GSEA

To assess the potential molecular mechanisms underlying our risk signature, GSEA was applied to identify enriched terms correlated with KEGG pathway in high-risk group. Significant gene sets were classified as those with normalized enrichment score (NES) > 1 and minimal *p* value < 0.05.

### 2.4. TIMER and ssGSEA

TIMER is an integrated website, which could measure immune infiltrate levels in various cancers, including ACC. In our study, we assessed the correlation between the hub ferroptosis-related genes with the contents of six immune cells, including CD4+ T cells, CD8+ T cells, B cells, neutrophils, dendritic cells, and macrophages in AGG via the TIMER. ssGSEA was performed to evaluate scores of 13 immune-related pathways and 16 immune cells in two risk groups.

## 3. Results

### 3.1. Construction of a Ferroptosis Risk Signature

**Figure 1:**
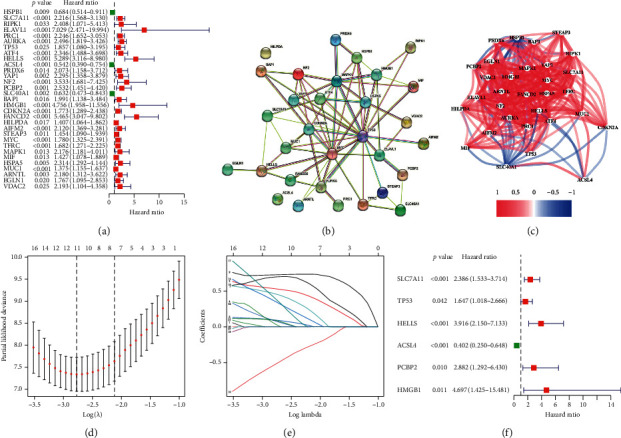
Construction of ferroptosis risk signature. (a) Forest plots showing the results of ferroptosis-related genes associated with OS by univariate Cox regression. (b) PPI network indicating the interactions among the candidate genes from the STRING. (c) The correlation network of candidate genes. (d) Partial likelihood deviance for the lasso regression. (e) Lasso profiles of ferroptosis-related genes. (f) The ferroptosis risk signature developed by multivariate Cox regression.

Flow chart of our research was displayed in Supplementary Figure S1. To construct a ferroptosis risk signature and explore its prognostic value in ACC patients, a total of 94 overlapping ferroptosis-associated genes derived from the TCGA and GEO database were preserved for further analysis. Then, univariate Cox regression analysis was performed to assess the association between the expression levels of these 94 genes with ACC clinical survival information in the TCGA dataset. We found 31 ferroptosis-associated genes correlated with OS of ACC patients (Figure 1(a), *p* < 0.05).  Figure 1(b) displayed the PPI network suggesting that TP53, HMGB1, CDKN2A, and MAPK1 were the hub genes. The association among these genes was exhibited in Figure 1(c). 11 ferroptosis-associated genes were finally reserved after lasso regression analysis (Figure 1(d)). In addition, through further multivariate Cox regression analysis to eliminate the possible interaction among the candidate genes, six genes (SLC7A11, TP53, HELLS, ACSL4, PCBP2, and HMGB1) were screened out (*p* < 0.05), suggesting their strong correlations to the OS of ACC patients (Figure 1(f)). The multivariate Cox regression coefficients and expression levels of the six ferroptosis-associated genes were used to construct a ferroptosis risk model. The formula for risk score calculation is as follows: risk score = (0.88 ∗ SLC7A11) + (0.50 ∗ TP53) + (1.37 ∗ HELLS) − (1.37 ∗ ACSL4) + (1.06 ∗ PCBP2) + (1.55 ∗ HMGB1).

### 3.2. Prognostic Performance of the Ferroptosis Risk Signature in TCGA

**Figure 2:**
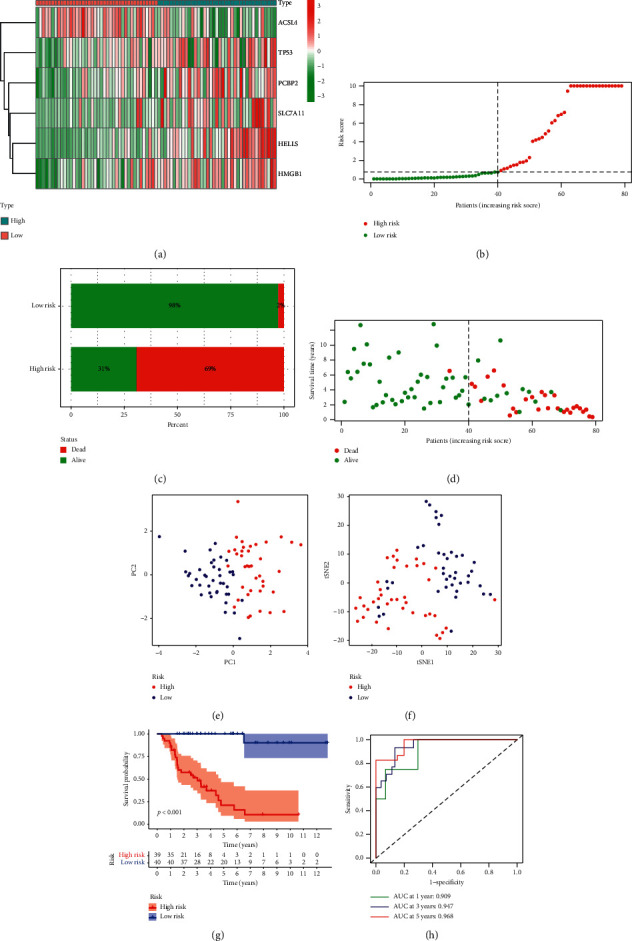
Prognostic value of the ferroptosis risk signature in TCGA. (a) Heatmap of the 6 ferroptosis-associated genes in two groups. (b) The distribution and median value of the risk scores. (c), (d) The mortality in two groups. (e) The PCA plot. (f) The t-SNE analysis. (g) K-M curves for the OS of patients in two groups. (h) ROC curve for assessing the predictive efficiency of the risk signature.

As seen in Figure 2(a), most of ferroptosis-associated genes were linked with a higher risk score in TCGA cohort. Based on the median risk score, individuals in TCGA were assigned to high-/low-risk group (Figure 2(b)). Patients in high-risk class had higher mortality than those in low-risk class (Figures 2(c) and 2(d)). PCA and t-SNE showed that individuals in disparate categories were distributed in different directions (Figures 2(e) and 2(f)). Furthermore, patients in high-risk group were inclined to have shorter OS than those in low-risk group (Figure 2(g)). In addition, the AUC in TCGA reached 0.909 at 1 year, 0.947 at 3 years, and 0.968 at 5 years, respectively (Figure 2(h)). These results suggested that the novel ferroptosis risk signature had a definite effect in predicting the prognosis of ACC patients. K-M curves for each of the six hub genes included in the risk model in ACC are demonstrated in Supplementary Figure S2, showing that lower expression of ACSL4 and higher expression of SLC7A11, TP53, HELLS, PCBP2, and HMGB1 were associated with poor survival. Considering heterogeneity of ACC patients, we further assess whether the ferroptosis risk signature had good performance in predicting the prognosis of ACC patients in different stages. As shown in Supplementary Figure S3, patients in high-risk group had shorter OS than those in low-risk group in both stage 1-2 group (*p*=0.00062) and stage 3-4 group (*p* < 0.0001), suggesting that our ferroptosis risk signature also performed well in predicting prognosis of ACC patients in different stage.

### 3.3. Validation of the Ferroptosis Risk Signature in GEO

**Figure 3:**
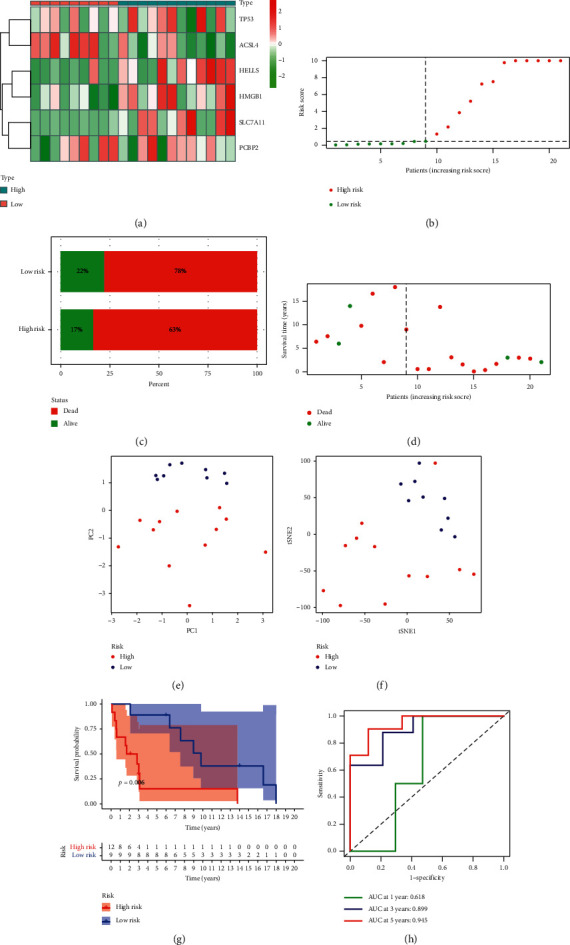
Validation of the ferroptosis risk signature in GEO. (a) Heatmap of the 6 ferroptosis-associated genes in two groups. (b) The distribution and median value of the risk scores. (c), (d) The mortality in two groups. (e) The PCA plot. (f) The t-SNE analysis. (g) K-M curves for the OS of patients in two groups. (h) ROC curve for evaluating the prognostic effect of the risk signature.

In GEO cohort, the heatmap revealed that most of the ferroptosis-associated genes were also related with a higher risk score (Figure 3(a)). In the same way, ACC patients in GEO were categorized into two groups (Figure 3(b)). Similarly, PCA and t-SNE analysis showed that patients in GEO with different risk scores were distributed in disparate directions (Figures 3(e) and 3(f)). As shown in Figure 3(g), the OS of patients in high-risk class was obviously shorter than that of patients in low-risk group in GEO cohort (*p*=0.006). In addition, the ROC curve was drawn to assess the predictive performance of the risk model. The AUC at 1-, 3-, and 5-year in GEO cohort were 0.618, 0.899, and 0.945, respectively, indicating that the risk signature had a favourable capacity in predicting prognosis of ACC patients (Figure 3(h)).

### 3.4. Independent Prognostic Value of the Ferroptosis Risk Signature

**Figure 4:**
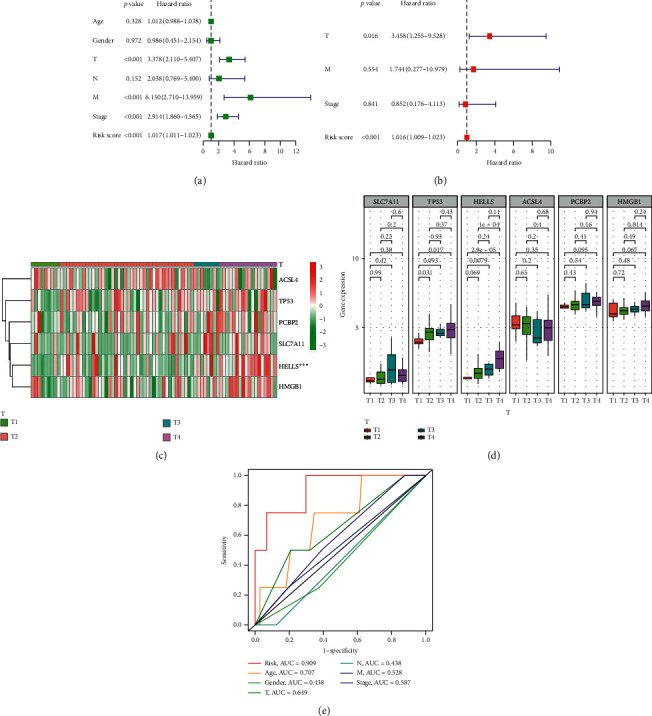
Independent prognostic value of the ferroptosis risk signature. (a), (b) Univariate and multivariate Cox regression analyses for evaluating the independent prognostic effect of the risk signature in TCGA cohort. (c) Heatmap of expressional profiles of 6 ferroptosis-associated genes at different T staging. (d) The expressional levels of 6 ferroptosis-associated genes at different T staging. (e) The AUC values of the risk factors.

We performed univariate and multivariate Cox regression analyses to observe whether clinical characteristics (such as age, gender, T, N, M, and stage) and the risk score are independent prognostic factors for OS. We discovered that the risk score and T staging were independent prognostic predictors for OS in TCGA cohort. Owning to incomprehensive clinical parameters, a further Cox regression was not conducted to assess the prognostic value in GEO cohort. In addition, we investigated the correlation between the six ferroptosis-associated genes with the pathological T staging in ACC patients. Heatmap showed the expressional profiles of the six ferroptosis-associated genes at different T staging in TCGA cohort (Figure 4(c)). As drawn in Figure 4(d), the expressional levels of the major ferroptosis-associated genes, except ACSL4, which was considered as a protective gene, were generally higher in ACC patients at advanced T staging. We further compared the prognostic efficiency of our ferroptosis risk signature with other common prognostic factors, including age, gender, T, N, M, and stage. As shown in Figure 4(e), our ferroptosis risk signature (AUC = 0.909) demonstrated significantly better prediction of ACC patients' OS at 1 year than age (AUC = 0.707), gender (AUC = 0.438), T staging (AUC = 0.649), N staging (AUC = 0.438), M staging (AUC = 0.528), and stage (AUC = 0.587). These results suggested that our risk signature performed better than other common prognostic characteristics.

### 3.5. GSEA for Identifying the Ferroptosis-Associated Signaling Pathways

**Figure 5:**
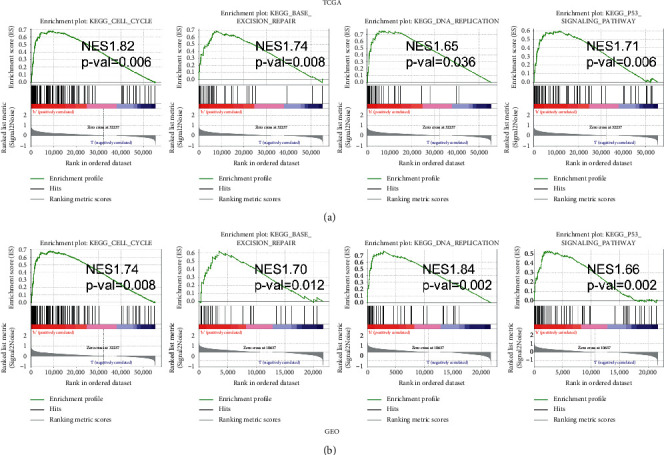
GSEA for identifying the ferroptosis-associated signaling pathways. (a) GSEA of the associated signaling pathway in TCGA cohort. (b) GSEA of the associated signaling pathway in GEO cohort.

We conducted GSEA to compare the biological signaling pathways between two groups. It was noteworthy that enriched gene sets linked to cell cycle, base excision repair, DNA replication, and P53 signaling pathway were highly enriched in high-risk group in both TCGA and GEO datasets (Figures 5(a) and 5(b).

### 3.6. Relationships between the Ferroptosis-Associated Genes and Immune Infiltration

**Figure 6:**
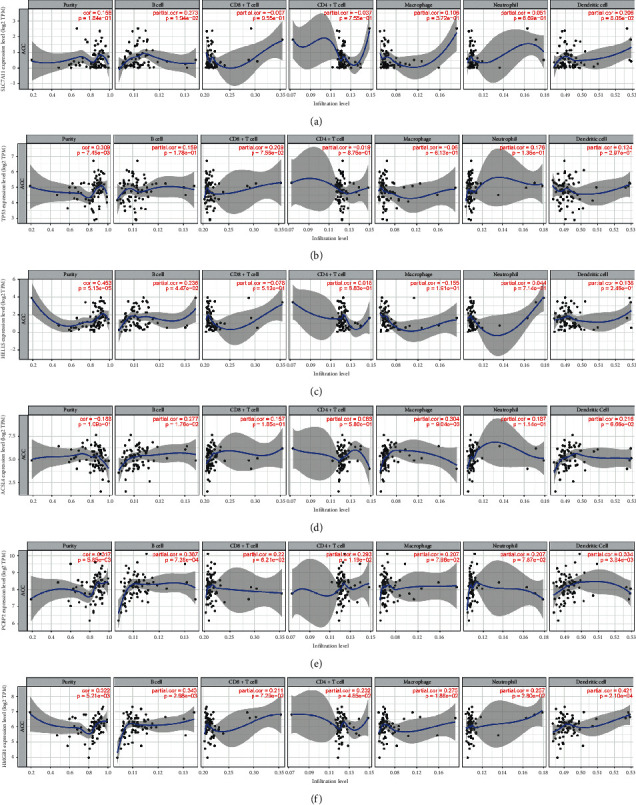
Relationships between the ferroptosis-associated genes and immune infiltration. (a–f). Relationship between the 6 hub ferroptosis-associated genes expression (SLC7A11, TP53, HELLS, ACSL4, PCBP2, and HMGB1) and the infiltrating levels of six immune cells in ACC.

Numerous studies demonstrated that the infiltration of cancer-related immune cells is associated with tumor development and prognosis. To identify whether there was a link between immune infiltration and the expressional levels of hub genes, we used TIMER to assess the correlation between the 6 hub genes and tumor purity along with six types of immune cells. As exhibited in Figure 6(a), we found the association between SLC7A11 expression and infiltration levels of B cells (*r* = 0.273, *p* = 1.94*e*−02), CD8+ T cells (*r* = −0.007, *p* = 9.55*e*−01), CD4+ T cells (*r* = −0.037, *p* = 7.55*e*−01), macrophages (*r* = 0.106, *p* = 3.72*e*−01), neutrophils (*r* = 0.051, *p* = 6.69*e*−01), and DCs (*r* = 0.206, *p* = 8.06*e*−02) in ACC. Besides, the other 5 hub ferroptosis-associated genes (TP53, HELLS, ACSL4, PCBP2, and HMGB1) included in the signature also showed significant correlation with the infiltrating levels of B cells (*r* = 0.159 to 0.387, *p* < 0.001), CD8+ T cells (*r* = −0.078 to 0.211, *p* < 0.001), CD4+ T cells (*r* = −0.019 to 0.293, *p* < 0.001), macrophages (*r* = −0.155 to 0.304, *p* < 0.001), neutrophils (*r* = 0.044 to 0.257, *p* < 0.001), and DCs (*r* = 0.206, *p* < 0.001) in ACC (Figures 6(b)–6(f)). In sum, these results revealed that these 6 hub genes were in varying degrees related to tumor-associated immune cells in the ACC microenvironment.

**Figure 7:**
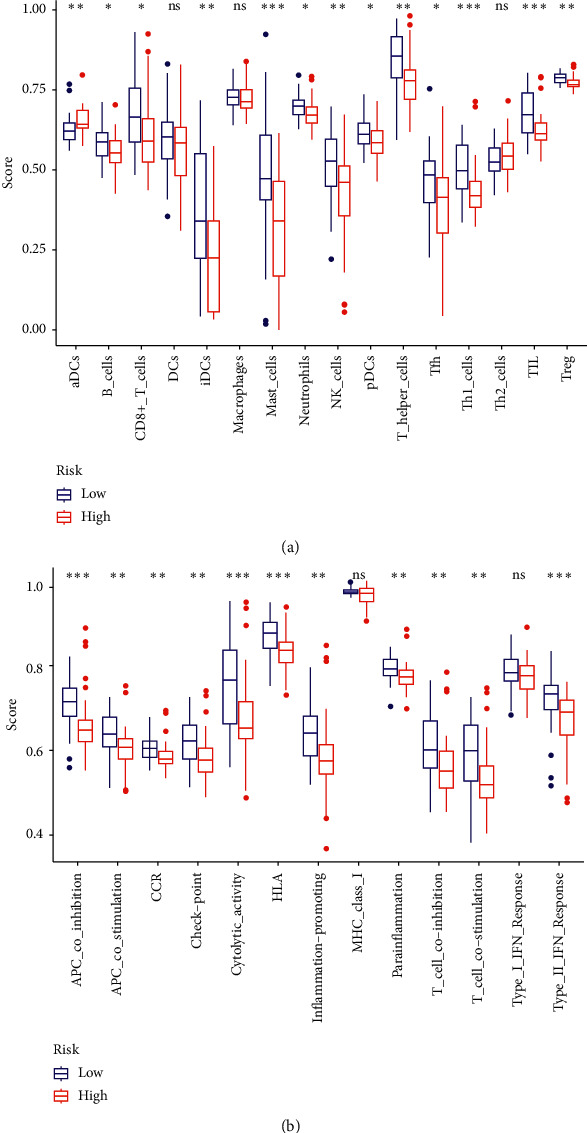
The ssGSEA scores of two groups in TCGA. (a) Scores of 16 immune cells. (b) Scores of 13 immune-related pathways.

To identify the association between our signature and immune microenvironment condition in ACC, we further performed ssGSEA to evaluate the scores of 16 immune cells and 13 immune-related pathways in TCGA cohort. We found that the contents of most immune cells in high-risk group, including aDCs, B cells, CD8+T cells, iDCs, mast cells, neutrophils, NK cells, pDCs, T helper cells, Tfh, Th2 cells, and Treg, were significantly lower than those in low-risk group (Figure 7(a)). Moreover, the scores of the most immune-related pathways were lower in high-risk group (Figure 7(b)). The above results suggested an immune suppressive microenvironment in ACC patients with high-risk scores.

## 4. Discussion

ACC is a rare endocrine malignancy with poor prognosis. For early diagnosis, more effective therapy, accurate prognosis of ACC, novel biomarkers, and prognostic signatures are required. Although there are some single gene and risk models linked to ACC patients' prognosis, no ferroptosis-related risk signature was reported for predicting prognosis in ACC. In our study, the associations between 103 ferroptosis-related genes and OS as well as immune cells infiltration were investigated in ACC patients. A new prognostic risk signature including six ferroptosis-associated genes correlated with immune cells infiltration was firstly constructed in ACC patients.

Ferroptosis, a novel form of cell death, emphasizes the importance of iron synthesis and metabolism, which was firstly proposed in 2012. On account of discovering the unique cell death form, numerous researches are focusing on the exploration of the potential mechanisms and therapy related to ferroptosis in multiple cancers. Previous studies show that ferroptosis also opens up a new potential avenue for cancer development and treatment [10, 11]. Furthermore, the expressional level of ferroptosis-related gene GPX4 and the sensitivity to ferroptosis were significantly increased in ACC, indicating that ACC patients may be susceptible to induction of ferroptosis [12]. In this study, we constructed a novel ferroptosis risk signature with powerful value in predicting ACC patients' prognosis for the first time. Furthermore, the risk signature had better prognostic efficiency than other common prognostic factors, including age, gender, T, N, M, and stage of ACC patients. The six ferroptosis-related genes adopted in the risk signature contained the risk-related genes (SLC7A11, TP53, HELLS, PCBP2, and HMGB1) and the protective gene (ACSL4).

These hub genes can be crudely categorized into four classes, including iron metabolism (PCBP2), lipid metabolism (ACSL4, HELLS), (anti)oxidant metabolism (SLC7A11, HMGB1), and cancer metabolism (TP53) [11]. Among these, tumor protein p53 (TP53) acts as an important tumor suppressor in cancer development and progression. Apart from the impact on apoptosis and cell cycle, TP53 could regulate cancer ferroptosis in a dual manner at transcriptional or posttranslational levels [13]. By targeting DPP4 and inducing P21 expression, TP53 could inhibit the ferroptosis. Conversely, ferroptosis could be enhanced by the inhibitory effect of TP53 on solute carrier family 7 member 11 (SLC7A11) in cancers [14]. Moreover, overexpression of SLC7A11, which had a high expression in several cancers, including ACC, inhibited the ferroptosis induced by ROS [14, 15]. Helicase lymphoid specific (HELLS, known as LSH), a chromatin remodeler, was shown to be linked with advanced stage and worse prognosis in pancreatic carcinoma, hepatocellular carcinoma, and nasopharyngeal carcinoma [16–18]. In lung carcinoma, Jiang et al. demonstrated that HELLS could inhibit ferroptosis by stimulating ferroptosis-associated genes SCD1 and FADS2 and lipid metabolism-related gene GLUT1 [19]. Poly(rC) binding protein 2 (PCBP2), an RNA-binding adaptor protein, could bind and deliver iron to ferritin for storage. Higher levels of PCBP2 are connected with worse prognosis in glioblastoma and gastric cancer [20, 21]. High mobility group box 1 (HMGB1), a nuclear protein, releases under the exposure to ferroptosis activators [22]. Ye et al. found that HMGB1 is a novel regulator of ferroptosis through RAS-JNK/p38 pathway in leukemia [23]. In addition, acyl-CoA synthetase long-chain family member 4 (ACSL4), a vital protein in ferroptosis, was overexpressed and served as an independent prognostic indicator in various cancers [24]. It is reported that ACSL4 is both a sensitive regulator and an effective inducer of ferroptosis [25]. In short, previous research revealed that these six genes are closely connected with ferroptosis and tumorigenesis, providing a powerful theoretical foundation for our risk model based on ferroptosis-related genes. Moreover, it was reported that ACC was associated with abnormal p53 signaling and frequent genetic alterations in TP53 [26]. In the study based on the comprehensive genomic characterization of 91 ACC patients, TP53 somatic alterations were reported to be the most frequent gene with genetic alterations [27]. TP53 p.R337H mutation is highly prevalent among children with ACC, accounting for 90% of ACC cases in Southern Brazil [28]. Although limited research focusing on the effects of these six hub genes on ACC have been published, we found that lower expression of ACSL4 and higher expression of SLC7A11, TP53, HELLS, PCBP2, and HMGB1 were related to poor OS of patients with ACC in our study, and the underlining mechanism needed further study. In addition, it is reported that adrenal cortex cells are extremely sensitive to ferroptosis due to their steroidogenic properties. Mitotane, as the only available drug applied in the treatment of ACC, is unable to induce ferroptosis [15]. Hence, it might be very promising in developing new drugs by inducing ferroptosis for ACC in years to come.

According to recent studies, ferroptosis could play a vital role in tumor immunotherapy [29–31]. IFN*γ*, released by CD8+ T cells, regulates lipid peroxidation and ferroptosis-related pathways in tumors. Several studies have shown that cells under the condition of ferroptosis could modulate anticancer immunity by releasing chemotaxis interacted with immune cells, such as NK and CD8+ T cells [30, 32]. Furthermore, a previous study reported that iron metabolism-related genes FPN1 and CP might participate in tumor immune microenvironment of ACC [33]. Of note, we mentioned that the ferroptosis-related genes in our signature have significant associations with immune cells, implying that ferroptosis and immunity in ACC microenvironment are complicated. Meanwhile, the lower scores of immune cells and immune-related functions in high-risk group suggested an immune suppressive microenvironment in ACC patients with high-risk scores, implicating that the poor prognosis of patients in the high-risk group might be caused by the immunosuppressive status. However, the underlying mechanisms between ferroptosis-related genes and tumor immunity in ACC remain poorly understood and warrant further investigation.

As far as we know, this is the first study aiming at constructing and validating a ferroptosis risk signature in patients with ACC. These results revealed that our ferroptosis risk signature could be considered as a powerful tool for predicting prognosis and reflecting the immune infiltration in ACC. However, there are several limitations in our study. Firstly, our study was retrospective based on public datasets, so further large-scale prospective researches and clinical trials are needed for validation of the prognostic ability. Besides, the mechanism of how ferroptosis modulates the development of ACC was not verified by functional experiments. Thus, further researches are required to confirm our findings and explore the underlying mechanisms before application in clinical practice.

## Data Availability

RNA-seq data and clinical information used to support the ﬁndings of this study were collected from the Cancer Genome Atlas (TCGA) (https://cancergenome.nih.gov/) and Gene Expression Omnibus (GEO) repository (GSE19750).

## Supplementary Materials

Supplementary Materials Figure S1: flow chart of the study. Figure S2: K-M curves for each of the 6 hub genes in ACC patients. Figure S3: Kaplan–Meier curves for patients assigned to high- and low-risk groups based on the risk score in TCGA cohort. Table S1: clinical characteristics of ACC patients in the TCGA and GEO. Table S2: 103 ferroptosis-related genes downloaded from the GeneCards.
