# Supplementary material for: Identification of a Ferroptosis-Related Signature Associated with Prognosis and Immune Infiltration in Adrenocortical Carcinoma
Source: Int J Endocrinol. 2021 Jul 20;2021:4654302. doi: 10.1155/2021/4654302 (PMC8318759; doi:10.1155/2021/4654302)
Supplement: Supplementary Materials — Figure S1: flow chart of the study. Figure S2: K-M curves for each of the 6 hub genes in ACC patients. Figure S3: Kaplan–Meier curves for patients assigned to high- and low-risk groups based on the risk score in TCGA cohort. Table S1: clinical characteristics of ACC patients in the TCGA and GEO. Table S2: 103 ferroptosis-related genes downloaded from the GeneCards. [file 4654302.f1.pdf]

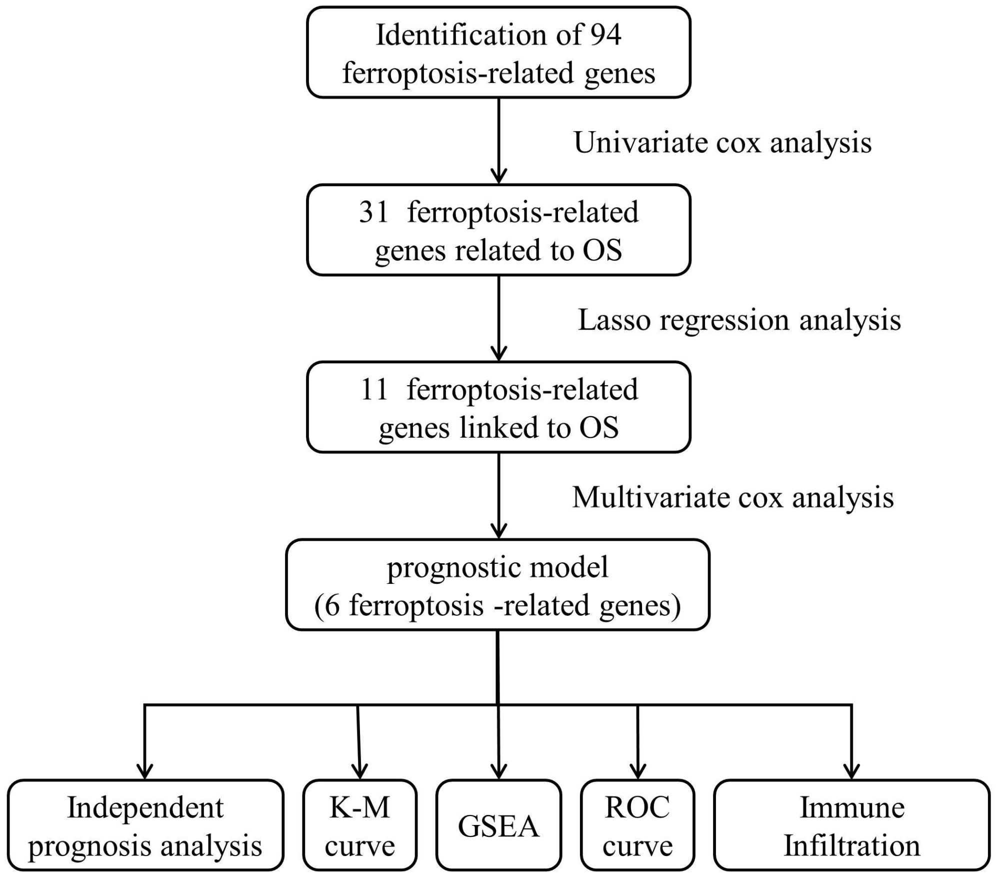

Figure S1. Flow chart of the study.

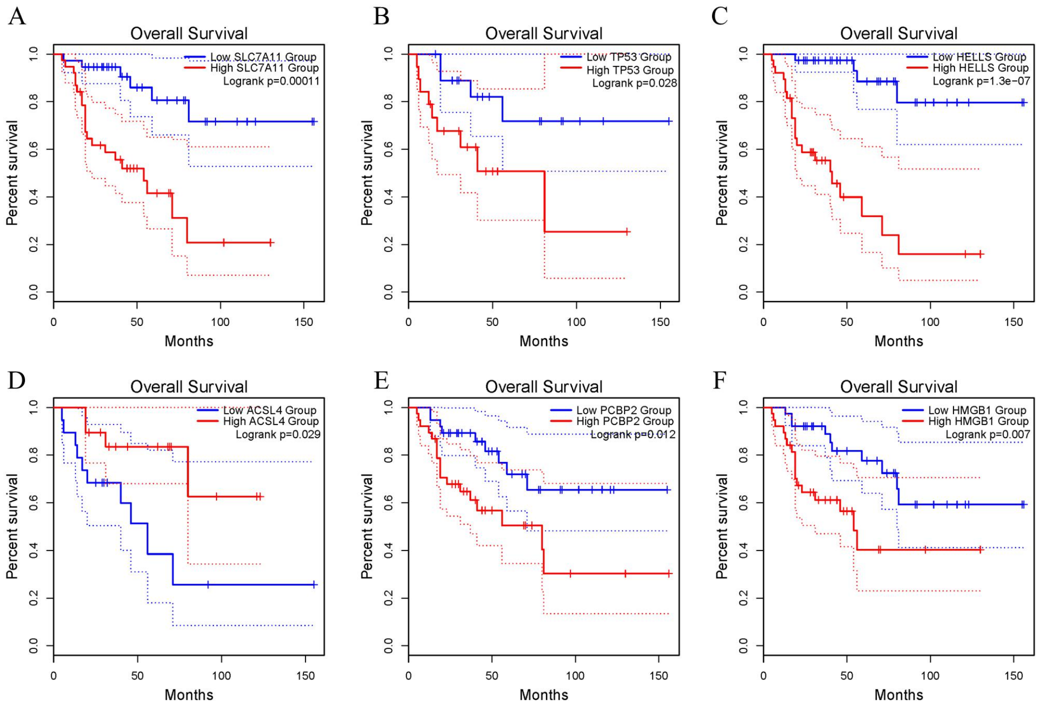

Figure S2. K-M curves for each of the 6 hub genes in ACC patients**.**

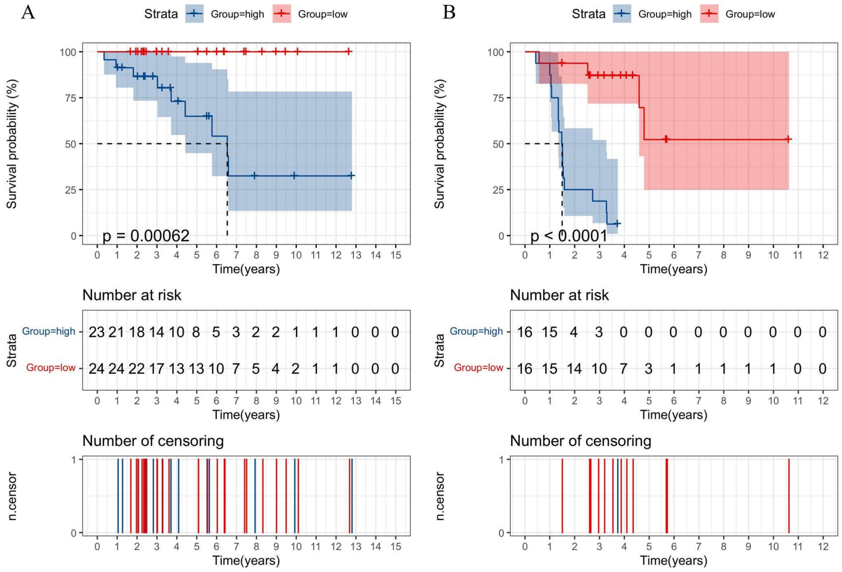

Figure S3. Kaplan-Meier curves for patients assigned to high and low risk groups based on the risk score in TCGA cohort.

A. K-M curve of patient in stage 1-2 group； **B.** K-M curve of patient in stage 3-4 group；

Table S1. Clinical characteristics of ACC patients in the TCGA and GEO.

Characteristics	Subgroup	TCGA(n=79)	GEO(n=21)
Age	≥50	39	14
	＜50	40	7
Gender	Feamle	49	10
	Male	30	11
Survival status	Dead	29	17
	Living	50	4
Stage	Stage1	9	1
	Stage2	36	7
	Stage3	16	1
	Stage4	16	4
	Recurrence	-	8
	unknown	2	-
T	T1	9	-
	T2	41	-
	T3	8	-
	T4	19	-
	unknown	2	-
N	NO	68	-
	N1	9	-
	unknown	2	-
M	M0	61	-
	M1	16	-
	unknown	2	-

Table S2. 103 ferroptosis-related genes downloaded from the GeneCards.

Gene Symbol	Description	Category
GPX4	Glutathione Peroxidase 4	Protein Coding
AIFM2	Apoptosis Inducing Factor Mitochondria Associated 2	Protein Coding
TP53	Tumor Protein P53	Protein Coding
ACSL4	Acyl-CoA Synthetase Long Chain Family Member 4	Protein Coding
SLC7A11	Solute Carrier Family 7 Member 11	Protein Coding
VDAC2	Voltage Dependent Anion Channel 2	Protein Coding
VDAC3	Voltage Dependent Anion Channel 3	Protein Coding
ATG5	Autophagy Related 5	Protein Coding
ATG7	Autophagy Related 7	Protein Coding
NCOA4	Nuclear Receptor Coactivator 4	Protein Coding
HMOX1	Heme Oxygenase 1	Protein Coding
SLC3A2	Solute Carrier Family 3 Member 2	Protein Coding
ALOX15	Arachidonate 15-Lipoxygenase	Protein Coding
BECN1	Beclin 1	Protein Coding
PRKAA1	Protein Kinase AMP-Activated Catalytic Subunit Alpha 1	Protein Coding
SAT1	Spermidine/Spermine N1-Acetyltransferase 1	Protein Coding
NF2	Neurofibromin 2	Protein Coding
YAP1	Yes1 Associated Transcriptional Regulator	Protein Coding
FTH1	Ferritin Heavy Chain 1	Protein Coding
TF	Transferrin	Protein Coding
TFRC	Transferrin Receptor	Protein Coding
FTL	Ferritin Light Chain	Protein Coding
CYBB	Cytochrome B-245 Beta Chain	Protein Coding
GSS	Glutathione Synthetase	Protein Coding
CP	Ceruloplasmin	Protein Coding
PRNP	Prion Protein	Protein Coding
SLC11A2	Solute Carrier Family 11 Member 2	Protein Coding
SLC40A1	Solute Carrier Family 40 Member 1	Protein Coding
STEAP3	STEAP3 Metalloreductase	Protein Coding
ACSL1	Acyl-CoA Synthetase Long Chain Family Member 1	Protein Coding
GCLC	Glutamate-Cysteine Ligase Catalytic Subunit	Protein Coding
MAP1LC3A	Microtubule Associated Protein 1 Light Chain 3 Alpha	Protein Coding
MAP1LC3B	Microtubule Associated Protein 1 Light Chain 3 Beta	Protein Coding
SLC39A14	Solute Carrier Family 39 Member 14	Protein Coding
SLC39A8	Solute Carrier Family 39 Member 8	Protein Coding
ACSL5	Acyl-CoA Synthetase Long Chain Family Member 5	Protein Coding
GCLM	Glutamate-Cysteine Ligase Modifier Subunit	Protein Coding
PCBP1	Poly(RC) Binding Protein 1	Protein Coding
PCBP2	Poly(RC) Binding Protein 2	Protein Coding
ACSL3	Acyl-CoA Synthetase Long Chain Family Member 3	Protein Coding

ACSL6	Acyl-CoA Synthetase Long Chain Family Member 6	Protein Coding
SAT2	Spermidine/Spermine N1-Acetyltransferase Family Member 2	Protein Coding
FTMT	Ferritin Mitochondrial	Protein Coding
LPCAT3	Lysophosphatidylcholine Acyltransferase 3	Protein Coding
MAP1LC3C	Microtubule Associated Protein 1 Light Chain 3 Gamma	Protein Coding
MAP1LC3B2	Microtubule Associated Protein 1 Light Chain 3 Beta 2	Protein Coding
BAP1	BRCA1 Associated Protein 1	Protein Coding
